# Brucellosis in hospitalized patients and their animals in the agro-pastoral region of Theniet El Had (Algeria) during ten years (2013−2023)

**DOI:** 10.1016/j.idcr.2024.e01945

**Published:** 2024-04-15

**Authors:** Wafa Ilhem Yahiaoui, Ali Dahmani

**Affiliations:** Institute of Veterinary Sciences, University of Saad Dahlab Blida 1, Road of Soumaa, BP 270, Blida 09000, Algeria

**Keywords:** Brucellosis, Patients, Animals, Algeria

## Abstract

**Introduction:**

Brucellosis is an important zoonosis problem worldwide. It’s also recognized as a clinical and health problem in Algeria.

**Methods:**

This research is a descriptive study to determine the prevalence of brucellosis and some clinical and epidemiological aspects of hospitalized patients in the agro-pastoral region of Theniet El Had for ten years, between March 2013 and March 2023. During the study period, 180 patients (61.66% men and 38.33% women) with confirmed brucellosis based on clinical symptoms and serological tests were hospitalized for treatment. Patients working with animals were requested to screen their animals (383 goats from 27 suspicious farms with no history of vaccination), 16, 71% of goats from 44%, and 44% herds were infected.

**Results:**

The occurrence of human cases varied from 49.18 to 66.02pcm (cases/100,000 inhabitants) with an average of 58, 48pcm (cases/100,000 inhabitants). Almost half of hospitalized people who had been in contact with animals could have been contaminated by direct contact. Consumption of unpasteurized dairy products during the last 2 months was at the order of 95.55%. A family history of Brucellosis was observed in 36 (20%) patients. Brucella epididymo-orchitis occurred in 18.01% while relapse occurred in a small proportion of 7.22%.

**Conclusions:**

Local authorities should prevent human brucellosis with surveillance systems, disease declarations, biosecurity programs, and warning communities about the hazards of consumption of unpasteurized milk and dairy products.

## Introduction

Brucella is one of the world’s major zoonotic pathogens caused by Brucellosis spp. and is a major concern of health services in endemic areas [1], particularly in Algeria [2], [3].

It is mainly acquired through direct contact with infected animals or by consumption of improperly cooked food of animal origin or by inhalation of animal secretions [4].

In endemic countries, physicians are familiar with the disease and the use of diagnostic tests for brucellosis is often routine [4].

The evolution of the number of new human cases reported is like that observed in goats, which constitute the most important reservoir of infection for humans in Algeria [3].

## Materials and methods

Patients data on Brucellosis hospitalized patients were collected from the regional hospital of Theniet El Had.

Theniet El Had is a rural and agro-pastoral region located in the north of Algeria.

During the period March 2013 to March 2023, 180 patients with confirmed brucellosis based on clinical symptoms and serological tests were hospitalized for treatment.

Brucellosis is a disease that can show a variety of symptoms and can be mistaken for other multi-system diseases. The diagnosis of brucellosis in suspected cases was performed by serological methods, including the Rose Bengal agglutination technique and Wright's reaction. Sensitivity and specificity of these tests compared to culture as the gold standard are 87.5% /100%, and 100% / 86.8% respectively [5], [6]. By using two serological tests, the study aims to avoid false-positive and false-negative results.The blood samples were collected simultaneously as brucellosis was diagnosed before the initiation of treatment. Patients diagnosed with brucellosis had a positive reaction to at least one test (Rose Bengal or Wright's reaction).

Brucellosis is a reportable disease in Algeria, with mandatory outbreaks in humans and animals. Patients working with animals were asked to screen their animals. 383 goats belonging to 27 farms with no history of vaccination against brucellosis and having been in contact with 27 hospitalized patients were sampled. Sera were analyzed by veterinarian authority using the Rose Bengal Agglutination Test and the complement fixation test for confirmation according to national protocol. All positive animals were slaughtered according to Algerian legislation.

## Results and discussion

### Incidence

We noticed an incidence of 180 human brucellosis during ten years in the region of Theniet El Had. This corresponds to a rate of 58, 48% (cases/100,000 inhabitants), varying from 49.18% to 66.02% (cases/100,000 inhabitants) during the study period (March 2013 to March 2023). In Algeria, the occurrence of human cases depends on the region varying from 9.89% to 65.87% (cases/100,000 inhabitants) [3].

### Gender

Gender distribution was 61.66% (111 /180 male against 38.33% (69 /180) female, which is in agreement with the higher seropositivity for men (59.45%) and women (40.54%) in the southern region of Sidi-Bel-Abbes [7]. In rural regions, men can be more interested in livestock activities and slaughtering.

### Age

The mean patient age was 36 years (range, 14–75 years). National Institute of Public Health for the general population found brucellosis more frequent in adults [8].

### Alimentation

Consumption of unpasteurized dairy products during the last 2 months was at the order of 95.55% (172/180). The main sources of brucellosis contamination in Algeria are the consumption of dairy products (86.22%) [8].

Consumption of unpasteurized dairy products is the most common route of digestive contamination in Algeria due to local alimentary habits where raw milk and its derivatives were highly valued (artisanal cheese, butter, whey, curdled milk, etc.), especially in traditional dishes [9].

### Corresponding family

A family history of Brucellosis was observed in 36 (20%) patients. Brucellosis causes socio-economic problems because of the incapacity to work during periods of illness. In Algeria, the expenditure for each patient is equivalent to eight months of the minimum interprofessional salary [9], this economic situation will be more serious it’s a simultaneous infection among family members.

### Genito-urinary complications

20 patients (out of 111 men hospitalized) with Brucella epididymo-orchitis (BEO) were admitted to Theniet el Had hospital during the period of study.

BEO was diagnosed based on the positive clinical (e.g. fewer, testicular pain or tenderness and scrotal swelling) and ultrasonographic findings of orchitis, in addition to a laboratory confirmation.

Brucellar epididymo-orchitis (BEO) is the major genitourinary complication of brucellosis [10], [11].

BEO occurred in 18.01% (20 /111) of infected men in Theniet el Had. Occurring BEO in about 2–14% of patients with brucellosis as a result of urine Brucella removal or sepsis [11].

Genitourinary (GU) complication requiring a long hospitalization and can lead to serious complications that may require orchiectomy if not treated on time. It requires special clinical attention in endemic countries.

### Relapse episode

Relapse occurred in a small proportion (7.22%, n = 13), and the majority (91.11%, n = 164) had a complete remission. All the relapsed patients recovered after the second course of antibiotic therapy.

To differentiate relapse cases from reinfection, we consider that reinfection is an acute/subacute form with positive blood cultures and/or high titer agglutination. Relapse cases are blood culture negative, may be serologically negative with Wright's reaction or may have low titer, but always come with clinical manifestations.

It is important to note that there is no association between the serum titer of antibodies and disease complications [12]. A low serological titer with no clinical manifestation cannot be used as a diagnostic factor for relapse cases.

The observed relapse rate in the present study corresponds to the literature, the relapse rate is 5–10% and the cure rate exceeds 80% when appropriate [13].

### Activity

30% (60 /180) of patients of patients reported that they had contact with animals. Contact with infected animals can be a source of contamination dermally, conjunctively, or aerially [14], [15]. Occupational exposure is an important source of brucellosis contamination in Algeria occurring in 42.06% [8].

### Animal investigation

64 goats were positive for brucellosis belonging to 12 herds giving an infection rate of 16,71% in goats and 44,44% in herds.

In Algeria, goats are an important reservoir of disease transmission [3]. A control program based on sanitary prophylaxis (Test and slaughter) was initiated in 1995 for cattle and goats however the program has not proven efficacy [6].

## Conclusion

Brucellosis is an important health problem in the agro-pastoral area of Theniet El Had. Brucellosis can have major social and economic consequences, especially in vulnerable rural populations with a family history of disease. Local authorities should further combat the endemic disease with surveillance systems, disease declaration, and awareness of the mechanisms of contamination. Preventing human brucellosis is necessary, through a biosecurity plan, and alerting the local population to consumption of pasteurized dairy products.

## Authors Agreement

Upon acceptance, the Authors: Wafa Ilhem YAHIAOUI and Ali Dahmani assign to the Journal the right to publish and distribute the manuscript in part or in its entirety.

Authors: Wafa Ilhem YAHIAOUI and Ali have approved the work for publication; have agreed to submit the article to the Journal; accept full responsibility for the content of the Article.

## CRediT authorship contribution statement

**Ali Dahmani:** Validation. **Wafa Ilhem YAHIAOUI:** Conceptualization, Data curation, Investigation, Methodology.

## Declaration of Competing Interest

The Authors: Wafa Ilhem YAHIAOUI and Ali Dahmani declare that there is no conflit of interest related to this research.
